# Screen time use in children under 3 years old: a systematic review of correlates

**DOI:** 10.1186/1479-5868-10-102

**Published:** 2013-08-23

**Authors:** Helena Duch, Elisa M Fisher, Ipek Ensari, Alison Harrington

**Affiliations:** 1 Department of Population and Family Health, Mailman School of Public Health, 60 Haven Avenue, B-2 Room 211, New York, NY 10032, USA; 2 University of Illinois - Champaign Urbana, 402 N Prairie St., Champaign, IL 61820, USA

**Keywords:** Infants, Toddlers, Screen time, Television, Sedentary behavior, Correlates, Children, Review

## Abstract

**Background:**

A large percentage (68%) of children under age 3 use screen media, such as television, DVDs and video games, on a daily basis. Research suggests that increased screen time in young children is linked to negative health outcomes, including increased BMI, decreased cognitive and language development and reduced academic success. Reviews on correlates of screen time for young children have included preschool age children and children up to age 7; however, none have focused specifically on correlates among infants and toddlers. As research suggests that screen media use increases with age, examining correlates of early media exposure is essential to reducing exposure later in life. Thus, this paper systemically reviews literature published between January 1999 and January 2013 on correlates of screen time among children between 0 and 36 months of age.

**Methods:**

Two methods were used to conduct this review: (1) Computerized searches of databases (PubMed, PsycINFO, ERIC, Medline); and (2) Reference sections of existing reviews and primary studies. Inclusion criteria were: (1) The article included separate data for children 36 months and younger, (2) English language, (3) peer reviewed article, (4) analysis reported for screen viewing as a dependent variable, (5) original research article and, (6) examined correlates or associations between screen time and other demographic, contextual or behavioral variables. Articles were compiled between 2011 and 2013 and evaluation occurred in 2012 and 2013.

**Results:**

The literature search identified 29 studies that met inclusion criteria. These studies investigated a total of 33 potential correlates, which were examined in this review. Findings suggest demographic variables most commonly correlated with high screen time among infants and toddlers are child’s age (older) and race/ethnicity (minority). Child BMI, maternal distress/depression, television viewing time of the mother and cognitive stimulation in the home environment were also associated with screen media use. Studies reported that child sex, first born status, paternal education, non-English speaking family, two-parent household, number of children in the home and non-parental childcare were not associated with screen time among children aged 0–36 months. Associations were unclear (fewer than 60% of studies report an association) for maternal age, maternal education and household income. The remaining correlates were investigated in fewer than three studies and thus not coded for an association.

**Conclusions:**

The correlates identified in this study point to avenues for intervention to reduce screen time use in young children. However, further research is necessary to explore a number of environmental, socio-cultural and behavioral correlates that are under-examined in this population and may further inform prevention and intervention strategies.

## Introduction

Television, DVDs and other forms of screen media are common pastimes among young children in the United States. Despite the fact that the American Academy of Pediatrics recommends that parents avoid exposing children 2 and under to screen media, a nationally representative survey found that 68% of children under the age of 2 use screen media in a typical day, and that average screen time was 2.05 hours per day [1]. In addition, children may be exposed to more time in front of the television in daycare (an additional hour per day) and home-based childcare settings [2].

Children from lower socio-economic backgrounds may experience disproportionately high rates of screen media time. A study of young children participating in the Women, Infants and Children (WIC) program in New York State found that 82% of one year-olds and 95% of two year-olds watched television and videos on a typical weekday [3]. The average amount of screen time increased with age. One year-olds spent an average of 10 hours per week watching TV/videos, while two year-olds spent approximately 15 hours per week watching TV/videos [3]. Additionally, of the total sample of 2 year-olds in this study, 43% watched more than 2 hours in a typical weekday. Other studies demonstrate that greater television watching in early childhood predicts increased television watching later in childhood [4].

Screen time use may have detrimental effects on children’s health and development [5-15]. Studies of young children report associations between screen time and cognitive development outcomes, such as short-term memory skills, academic achievement in reading and math, and language development [5,11,14]. High levels of screen time in early childhood also appear to negatively impact academic and social outcomes in the long-term [9]. Furthermore, while evidence for an association between screen time and BMI among preschool children was inconclusive [16], several studies have reported positive associations later in childhood [3,6,17,18]. Even background television exposure has been shown to impact development by reducing the amount and quality of interactions between parents and children [19-22]. Beyond the amount of screen time, the content of media exposure is associated with children’s developmental outcomes [5,11,23].

Excessive screen time has proven to be an unhealthy habit that begins to develop in early childhood [4]. However, little is known about correlates of screen media use for children under 3. Previous reviews aggregated these correlates with data from children older than 3 [16,24], but developmental differences in the infant/toddler years versus later childhood years make it important to examine this youngest age group separately. Infants and toddlers largely depend on their parents for accessing media and alternate activities, in contrast to older children who can more easily express activity preferences and make decisions about their daily activities. Understanding correlates for this age group will help inform clinical and educational practices in the development of early interventions to prevent excessive screen time and potentially the adverse health and developmental outcomes associated with it, particularly among high-risk groups.

Using a bioecological theoretical framework [25], this paper summarizes the literature examining correlates of screen media use in children 36 months of age and younger, with an emphasis on parental and home-based characteristics. Bioecological theory posits that child development is influenced by different environmental systems from the most proximal, including the biological make-up of the child, to the family, school, community and culture [25]. We use this framework to examine the influence of correlates of screen time at different environmental levels.

## Methods

A systematic review of the literature was conducted of articles published from January 1990 to January 31, 2013. Keywords included in the search were: early childhood, infant, toddler, television viewing, sedentary behavior, physical activity, screen media, correlates, inactivity, television, TV, video, computer, videogames. More specifically, the terms “early childhood”, “infant”, and “toddler” were individually searched in conjunction with each of the following items: “television viewing”, “sedentary behavior”, “physical activity”, “screen media”, “inactivity”, “television”, “TV”, “video”, “computer”, and “videogames”. The term “correlates” was added to these paired searches. Two methods were used to conduct the review: (1) computerized searches of the following databases: PubMed, PsycINFO, ERIC, Medline and (2) reference sections of existing reviews and primary studies were scanned for additional articles meeting criteria. Inclusion criteria were: (1) The article included separate data for children 36 months and younger, (2) English language, (3) peer reviewed article, (4) analysis reported for screen viewing as a dependent variable, (5) original research article and, (6) examined correlates or associations between screen time and other demographic, contextual or behavioral variables. Although the majority of articles used a measure of television viewing as screen time, we also included articles that measured computer use (the use of a computer for purposes other than playing videogames) and videogames (in the computer or other handheld device) as separate or aggregated measures of screen time.

After removing all duplicates, two independent reviewers screened all articles and filled out an inclusion/exclusion criteria table. When a disparity was identified, a third reviewer screened the article. Each article included in the review was summarized in a table including basic study characteristics (sample, methods, outcomes) and major findings. This table was further summarized to include a list of identified correlates by study and the direction of coded associations.

### Risk of bias assessment

A modified version of the Downs and Black [26] checklist was used to assess risk of bias in the studies included in this review. The checklist consists of 27 items, 10 of which (1–3, 6, 7, 10–12, 18, and 20) were relevant to the studies included in this review, resulting in a maximum possible count of 10 points (higher scores indicate superior quality). The risk of bias assessment was carried out by two independent assessors; when disagreements occurred, consensus was achieved through discussion.

### Coding of variables

The coding of variables followed the model used in previous reviews by Sallis et al. [27] Hinkley et al. [16], and Hoyos et al. [24] in which the association between a correlate and screen viewing was determined by dividing the number of associations supporting a relationship by the total number of studies that examined the variable. Findings were coded as positive (+), negative (−), or as non-associations (NA) when studies reported that no association was found between the variable and screen time. As the model suggests, we focused solely on consistency of associations and not on the strength of these associations [24]. Following the model of Hoyos et al., associations were coded as strong (S) when 75-100% of studies reported the given association (or non-association), moderate (M) for 60-74% of studies, and unclear (U) when fewer than 60% of studies supported the given association or non-association [24]. Consistent with previous reviews in this area, the associations were only reported on variables that were studied on three or more occasions [24,27]. For variables studied on fewer than three occasions, the association was identified as Inconclusive (IN) See Table 1.

**Table 1:** Rules for classifying associations between variables and screen media exposure

**Association codes**	**Meaning of code**	**% of studies examining variable needed to draw conclusion**
(+)	Positive association	60% or greater
(−)	Negative association	60% or greater
NA	No association	60% or greater
U	Unclear association	Less than 60%
Consistency codes		
S	Strong consistency	70-100% of studies examining given variable support one association
M	Moderate consistency	60-70% of studies support one association
IN	Inconclusive	Fewer than 3 studies examined variable, no conclusions can be drawn

* = 4 or more studies support the given association/non-association.

## Results

### Study characteristics

**Figure 1:**
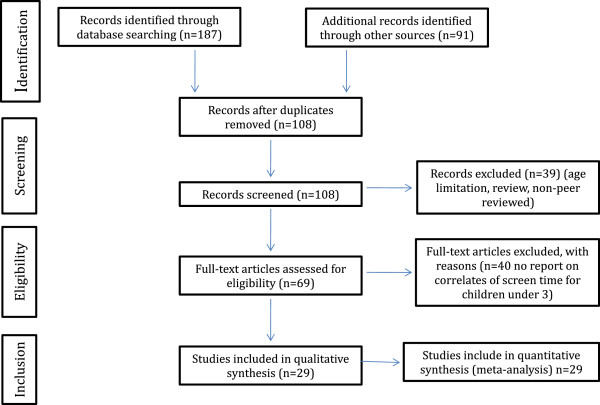
PRISMA flow chart.

The PRISMA statement [28] flow chart was used to track inclusion/exclusion of articles. One hundred and eight articles were identified after removing duplicates. Seventy-nine were excluded because: a) they did not meet the age criteria or did not report age separately (n = 15); b) they were a review paper or commentary (n = 18); c) they did not report on correlates of screen time (n = 40); or d) the article came from a non-peer reviewed source (n = 6). In total, 29 studies met inclusion criteria and were included in the quantitative review [3,4,8,10,12-15,17,23,29-47] (See Figure 1).

From the 29 studies included, 34% (n = 10) were published between 1999-2005 [3,4,10,13,14,23,29,33],[35,47] and 66% (n = 19) between 2006–2013 [5,8,12,15,17,30,32,34],[36-46]. Sixty-two percent (n = 18) of studies were cross-sectional [3,10,13,15,17,29-31,33,34],[36,37,39-41,45-47], 17% (n = 5) were longitudinal [23,32,42-44], 17% (n = 5) included both cross-sectional and longitudinal analyses [4,12,14,35,38], and 3% (n = 1) were randomized controlled trials [8]. The majority of studies (83%, n = 24), relied on parental report via interview or survey as a measure of screen media use [3,4,10,12-15,17,29,30,32-34],[36,38-47], while 17% (n = 5) [8,23,31,35,37] of studies utilized verbal or written time use diaries to assess total screen time. Eighty-six percent of studies (n = 25) were conducted with US samples [3,4,8,10,12-15,17,23,29-31],[33-35,37,38,40,41,43-47], 7% (n = 2) with European samples (Greece) [36,39] and 7% (n = 2) with Asian samples (Japan and Thailand) [32,42]. Of the studies conducted in the United States, 40% (n = 10) included nationally represented samples [4,10,13,14,17,29,37,45-47]. Sample size of 0–36 month-olds disaggregated from other ages varied: 14% (n = 4) of studies had a sample size between 1 and 99, [12,23,30,40] 41% (n = 12) had sample sizes between 100–999 participants [8,13,31-36,42-44,46], 28% (n = 8) between 1000–1999 participants [3,14,15,17,29,37,38,45], and 17% (n = 5) with sample sizes of over 2000 participants [4,10,39,41,47]. Two studies reported only total sample size, which included children above 36 months of age and offered between group comparisons between 0–36 month-old children and older children, with age being the only correlate reported. All articles included television viewing as a measure of screen time. Forty-one percent (n = 12) included only television [4,10,14,23,30,32,34,35],[37,42,44,47],, while 62% (n = 17) included television and videos/DVDs [3,12,13,15,17,29,31,33],[36,38-41,43,45,46,48], of which 29% (n = 5) also other forms of electronic media, such as computer games, e-books and videogames [8,12,13,29,46]. Only one study (3%) reported reliability data for their screen time measure [34]. Another study reported pilot-testing but not validating questions [3].

### Correlates of screen time for children under 3 years

Table 2 presents a summary of all correlates studied and their association with screen media use. Results are presented using a bio-ecological framework, from the most proximal environmental influences to the most distant [25].

**Table 2:** Summary of correlates

**Variable type**	**Variable**	**Positive association**	**Negative association**	**No association**	**Association (% of studies examining variable that support association)**	**Consistency strength**
**Child biological and demographic factors**						
	Sex	[35]		[3,4,12,14,35,36,38,43],[46]	NA (9/10 = 90%)	S*
	Child’s age	[3,4,10,12,14,15,17,23],[29,33,36,39,40,42,43]	[37]_a_	[12,31]	(+) (15/17 = 88%)	S*
	Ethnicity/race (non-caucasian)	[3,4,8,15,17]_b_[34,43,47],	[17]_c_	[14,46]	(+) (8/11 = 73%)	S*
	First born			[4,12,36]	NA (3/3 = 100%)	S
	BMI	[3,36,38,43]			(+) (4/4 100%)	S*
**Family biological/demographic factors**						
	Maternal age		[33,38,40]	[12,14,34,44]	U (4/7 = 57%)	
	Maternal education		[4,34,36,38,43]	[3,12,13,15,31,44,46]	U (7/12 = 58%)	
	Paternal education		[15]	[4,14]	NA (2/3 = 67%)	M
	Parental education (data not disaggregated)		[3]		IN	
	Maternal employment (employed)			[4,14,34,36,44,46]	NA (6/6 = 100%	S*
	Paternal employment (employed)			[4]	IN	
	Household income		[17,30,32,33,43]	[4,14,15,31,46]	U (5/10 = 50%)	
	Maternal BMI (Obese)	[44]			IN	
	Language (non-english speaking)		[17]_c_	[12,14,43]	NA (3/4 = 75%)	S
	Mother non-US born	[12]_f_		[12]_g_	IN	
**Family structure variables**						
	Two parent household		[38]	[12,15,43,44,46]	NA (5/6 = 83%)	S*
	Number of children in home		[15]_d_	[14,36,43]	NA (3/4 = 75%)	S
**Sociocultural/environmental factors**						
	Cognitive stimulation at home (as measured by HOME or StimQ)	[4]_e_	[8,38]		(−) (2/3 = 67%)	S
	Distressed/depressed mother	[30,34,38,41,45]		[4,43,44]	(+) (5/8 = 63%)	M*
	Non-parental child care		[4]	[12,14,15,38]	NA (4/5 = 80%)	S*
	Lives in urban area	[36]_h_		[14]	IN	
	Season (winter)	[31]			IN	
	Television in bedroom	[3,46]			IN	
	Parent belief child enjoys TV	[33,40]			IN	
	Parent belief in educational value of TV	[33,46]			IN	
	Heavy TV use in the home (includes background television)	[13]			IN	
	Shorter breastfeeding	[43]			IN	
	TV viewing time of father	[36]			IN	
	TV viewing time of mother	[36,43,44]			(+) 3/3 = 100%	S
	Time/content restrictions			[31]	IN	
**Behavioral factors**						
	Daily sleep duration of child		[43]		IN	
	Infant crying duration	[44]				
	Onset age of watching TV	[4]			IN	

a = background television; b = for children 12–23 months of English-speaking Latino mothers only, c = for children 24–35 months of Spanish-speaking, Latino mothers only, d = association only for 2 or more siblings; e = for infants 0–11 months, f = at 33 months only; g = at 21 months only; h = weekends only.

(+) = positive association.

(−) = negative association.

NA = studies consistently report no association.

U = Unclear association (fewer than 60% of studies support any association or non-association).

(S) = Strong consistency (70-100% of studies support the reported association or non-association).

(M) = Moderate consistency (60-70% of studies support the reported association or non-association).

* = 4 or more studies support the given association/non-association.

IN = inconclusive (variable not studied on 3 or more occasions).

(Dennison et al., [3]), (Certain & Khan, [4]), (Mendolsohn et al., [8]), (Thompson & Christakis, [10]),(Tomopoulos et al., [11]), (Tomopoulos et al., [12]), (Vandewater et al., [13]), (Zimmerman & Christakis, [14]), (Zimmerman et al., [15]), (Thompson et al., [17]), (Linebarger, & Walker, [23]), (Anand & Krosnick, 2005[29]), (Bank et al., [30]), (Barr, et al., [31]), (Cheng, et al., [32]), (Dalzell, et al., [33]), (Horodynski et al., [34]), (Huston, et al., [35]), (Kourlaba et al., [36]), (LaPierre et al., [37]), (Lumeng et al., [38]), (Masur & Flynn, [40]), (McLearn et al., [41]), (Ruangdaraganon et al., [42]), (Schmit et al., [43]), (Thompson et al., [44]), (Thompson & Christakis, [45]), (Vandewater et al., [46]), (Flores et al., [47]).

#### Child biological and demographic factors

Positive associations were identified in studies reporting a relationship between screen time and child’s age. Among the 17 studies that included age as a correlate, the majority (15 positive associations/17, 88%) report that older children watched more television than younger children [3,4,10,12,14,15,17,23],[29,33,36,39,40,42,43]. A positive association was also consistently found between screen media exposure and membership in a racial/ethnic minority group (8 positive associations/11, 73%) [3,4,15,17,34,43,47,48] and child body mass index (BMI) (4 positive associations/4, 100%) [3,36,38,43]. Studies consistently reported no association between screen time and child’s sex (9 no associations/10, 90%) [3,4,12,14,35,36,38,43],[46] or first-born status (3 no associations/3, 100%) [4,12,36] (See Table 2).

#### Family biological and demographic factors

No association was identified between screen time and maternal employment (6 no associations/6, 100%) [4,14,34,36,44,46], family’s native language (non-English speaking) (3 no associations/4, 75%) [12,14,43] or paternal education (2 no associations/3, 67%) [4,14]. Associations are unclear between screen time and maternal age (4 no associations/7, 57%) [12,14,34,44], maternal education (7 no associations/12, 58%) [3,12,13,15,31,44,46] and household income (5 no associations/10, 50%) [4,14,15,31,46]. Other correlates, such as maternal BMI, paternal employment and maternal country of origin, were examined in fewer than 3 studies and associations cannot be reported.

#### Family structure factors

The number of children in a home (3 no associations/4, 75%) and living in a two-parent household (5 no associations/6, 83%) [12,15,43,44,46] were not associated with screen media exposure in children under 36 months.

#### Behavioral factors

The time a mother spends watching television was positively associated with the child’s viewing time (3 positive associations/3, 100%) [36,43,44]. All other behavioral correlates (sleep duration of the child, infant crying duration, onset age of watching TV, TV viewing of father, and time/content restrictions on screen media) were examined in fewer than 3 studies and remain inconclusive.

#### Sociocultural/environmental factors

Increased access to cognitive stimulation in the home (as measured by either the StimQ [8] or the HOME [49]) was negatively associated with screen media exposure among children 0–36 months (2 negative associations/3, 67%) [8,38]. The HOME and the Stim Q measure the quality and quantity of stimulation and support available to a child in the home environment (e.g. availability of educational toys, time parent spends reading, etc.). Maternal distress/depression was examined in 8 studies using well characterized measures [the Center for Epidemiological Studies Depression Scale (CES-D) [4,30,34,38,41,44], the Edinburgh Post-partum Depression Scale [43] or the Mental Health Inventory 5 (MHI-5) [45]] and was positively associated with screen time (5 positive associations/8, 63%) [30,34,38,41,45].

No association was found between screen time and child participation in non-parental child care, such as day care or Early Head Start (4 no associations/5, 80%) [12,14,15,38]. Of the other 6 sociocultural/environmental variables explored by at least one study, none were examined with enough frequency to draw conclusions. These included living in an urban environment, seasonality, having a television in the child’s bedroom, heavy television use in the household and parental beliefs regarding the value and enjoyment of television for their children.

## Discussion

This review summarizes literature related to correlates of screen media use in children age birth to 36 months. Two previous reviews focused on screen time correlates for young children: one specifically focused on preschool-aged children [16] and the other on children under age 7 [24]. Neither disaggregated data for infants and toddlers. Our findings share consistencies with these reviews but discrepancies are clear. Consistent with Hinkley [16], our review of correlates to screen viewing for children under 36 months identified no association between child’s sex, the presence of siblings and screen time, and an unclear association with regard to maternal education. Discrepant with Hinkley et al. [16], our review identified an association between child age, child body mass index, race/ethnicity (minority) and screen time among infants and toddlers.

Our results more consistently matched those of Hoyos and colleagues’ [24] likely because infants and toddlers were included in their review whereas Hinkley’s [16] data focused solely on preschoolers. Consistent with Hoyos et al. [24], our review identified positive associations in the demographic and anthropomorphic variables above mentioned, as well as inconclusive associations with family origin. However, discrepant from their review, neither family structure variable (two parent household and number of children in the home) was associated with screen viewing for infants and toddlers.

Three variables (maternal age, maternal education and household income) yielded unclear associations as studies were split between a negative association and no association. A lack of consistent comparison groups (i.e. heterogeneous versus homogeneous samples) for these variables may account for these results. Furthermore, the lack of uniformly defined variables and cut points to examine the impact of these variables on screen time may have contributed to inconsistencies.

Maternal employment (employed), two-parent household, number of children in the home and non-parental childcare were consistently not associated with screen time use in infants and toddlers. Similarly, Hinkley et al [16] identified no association between parental employment, the presence of siblings and screen time for preschool children. This finding contradicts the hypothesis that caregivers’ work schedules and lack of childcare significantly contribute to the increased use of screen media in children and warrants further investigation to explore the role of family structure and childcare in facilitating or hindering the use of media in young children.

Maternal distress/depression was moderately positively associated with screen time. Most studies used the Center for Epidemiological Studies-Depression Scale (CES-D), a well-characterized measure of depression risk in the general population. The children in these studies ranged from 3 to 33 months with no clear pattern to differentiate between those studies that identified an association from those that did not. Thus, further exploration of maternal depression, with careful consideration to children’s age, timing of depression measurements and consistency of covariates, is warranted. In addition, the mechanisms by which maternal depression may impact screen time are largely unknown and should be further explored.

A moderate inverse association was found between screen time and cognitive stimulation in the home (as measured by HOME/StimQ). Cognitive stimulation includes a variety of activities and materials that require parent support and engagement, often used as a proxy for parenting support and stimulation to the child [8,49]. Certain and colleagues [4] found that cognitive stimulation, as measured by the HOME short form, was inversely associated with screen time for older toddlers (24–35 months) in bivariate analysis but positively correlated with screen time for younger infants (0–11 months) in multi-variable analysis. The authors hypothesized that parental beliefs about the positive impact of TV on their children’s language may account for this finding. Two studies in our review support this hypothesis, having both found a positive association with parental beliefs in the educational value of screen media, [33,46] despite the lack of conclusive evidence in this area.

The other two studies that examined cognitive stimulation in the home found that diminished cognitive stimulation was associated with increased screen media use. Similarly, Mistry et al. [50] conducted a study with preschoolers and found that high screen media use was associated with low levels of stimulation in the home and with low parental involvement [50]. Randomized control trials conducted by Mendelsohn et al. [8], and Dennison et al. [51] support the finding that parent–child interactions and parental stimulation in the home play a role in young children’s screen time exposure. Both studies examined interventions aimed towards increasing parent–child interaction and stimulation and reported significantly reduced screen time among the intervention group compared to a control group [8].

### Limitations

While some studies reported data separately for infants and toddlers, this was not consistently done, making it difficult to separate correlates for the two age groups. There are vast developmental differences between the infant and toddler years and the AAP recommendations set different guidelines for each age group. Thus identified correlates may also vary at different ages. For example, LaPierre and colleagues found that children aged 8–24 months are exposed to more background television than older children [37], while our review reports that older children consistently watch or are intentionally exposed to more overall television.

Similarly, while all studies examined television as screen time, only five studies examined videogames and computer games. Therefore, we were not able to report separate results for different kinds of media. Though new technologies and media use are prevalent at younger ages, we hypothesize that different age groups are more likely to access different types of media (e.g. a baby is more likely to be placed in front of a television or DVD while a toddler may interact more actively with a videogame), and correlates may not be equally supported in each age group.

Research has consistently identified the content of media use to be an essential factor to consider in the study of children’s screen time [5,11,23]. However, we did not have enough information in this review to examine how media content is associated with media use in young children.

Over half of the studies (62%) included in this review were cross-sectional making it difficult to establish casual relationships between correlates and screen time. Of the remaining studies, many of the findings limited to infants and toddlers were found only in cross-sectional analyses, further limiting inference on causal conclusions. Additionally, over 50% of studies have samples >1000 subjects, allowing possibly trivial variables to appear statistically significant when they may be inconsequential.

Heavy reliance on parental report as a measure of screen time is a major limitation of the literature due to the risk of recall and social desirability biases. Most studies (83%) used parental report as a measure of screen time use; only one used a scale that had validated television use via observation [34]. While some studies attempted to control for bias by using verbal or written screen time diaries, others simply asked parents to report on children’s screen time in a typical day, resulting in extreme variation within the screen time exposure construct. Such variation likely explains some of the inconsistencies in our results.

Consistent with previous reviews [16,24], this study does not report on the variables’ effect sizes but only on the consistency of the association between a variable and screen/media use in the review of the literature.

### Future directions

Research is needed to develop objective measures for screen media use in young children and to establish the reliability and validity of these measures. Some physical activity measures, like the Behaviors of Eating and Activity for Children’s Health (BEACHES) [52] and the Observation System for Recording Physical Activity in Children home version (OSRAC-H) [53] include observational time samples of children’s media use. However, these instruments have not been validated for use with infants and toddlers.

Further research is also necessary to distinguish between correlates for infants (under 12 months) and toddlers, as well as different kinds of media (TV, DVD, videogames, computer games) and media content. This will help identify distinct age, content and media type correlates that could guide intervention.

Several correlates in this review had unclear associations with screen time (e.g. maternal age, maternal education, household income). Further research should focus on clearly defining these constructs and exploring their relationship to screen time use. Other correlates such as maternal depression and cognitive stimulation in the home, were only moderately associated with screen time. These modifiable correlates should be further studied to elucidate their role and the mechanisms by which they may impact young children’s screen habits.

Similarly, a series of behavioral and environmental factors, such as daily sleep duration, infant crying duration, breastfeeding duration, TV time and content restrictions, were studied in fewer than three occasions but point to other modifiable constructs that may provide additional opportunities for intervention.

Finally, there are a number of large longitudinal datasets that include information on screen time use for young children. Studies that compare data from different datasets with well-defined and comparable populations, outcomes, predictors and covariates can help clarify some of the inconsistencies identified in the literature and significantly inform the design of interventions to reduce screen media use in infants and toddlers. Interventions well-grounded in research evidence may have a lasting impact in the screen time habits of the youngest children.

## Conclusion

This review identifies demographic, environmental, socio-cultural and behavioral correlates to screen time use in infants and toddlers. These correlates can help target at risk groups and provide insight into the design of prevention and intervention strategies to reduce screen time use in young children. The review also identifies significant areas for further research, particularly in the development and use of reliable measures of screen time and design of studies with well-defined, comparable variables that can help unpack inconsistencies in the existing literature.

## Competing interests

The authors declare that they have no competing interests.

## Authors’ contributions

HD conceptualized and designed the review, supervised the analysis, provided overall quality assessment and drafted the manuscript. EF conducted the analysis, was involved in quality assessment, interpretation of results and assisted with drafting the manuscript. IE and AH participated in the data extraction and quality assessment, as well as interpretation of results. All authors read and approved the final manuscript.
